# Rapid whole genome sequencing in newborn screening for metabolic diseases

**DOI:** 10.3389/fped.2025.1686738

**Published:** 2025-12-01

**Authors:** Jun Zheng, Xin Yang

**Affiliations:** Neonatal Disease Screening Center, Huai'an Maternal and Child Health Care Hospital Affiliated to Yangzhou University, Huaian, Jiangsu, China

**Keywords:** rapid whole genome sequencing, metabolic diseases, newborn screening, precisionmedicine, NICU diagnostics

## Abstract

**Background and purpose:**

Metabolic disorders, which are estimated to include approximately 1,500 distinct conditions such as urea cycle disorders, lysosomal storage diseases, and mitochondrial dysfunctions, pose a significant clinical challenge due to their genetic heterogeneity and rapid onset of symptoms in newborns. Delays in diagnosis often lead to irreversible damage or mortality. Rapid whole genome sequencing (rWGS) has emerged as a transformative diagnostic tool, offering comprehensive genetic insights within 24–72 h.

**Materials and methods:**

This study reviews the application of rWGS in the early detection and management of metabolic diseases, emphasizing its role in overcoming limitations of traditional diagnostic methods.

**Results:**

The integration of rWGS into clinical workflows offers a high diagnostic yield, exceeding 50% in neonatal intensive care units (NICUs), where timely interventions are critical. Utilizing advanced sequencing platforms, such as Illumina NovaSeq and Oxford Nanopore, coupled with optimized bioinformatics pipelines, rWGS enables precise variant identification and prioritization. Key findings highlight its capacity to accelerate diagnosis, inform therapeutic decisions, and reduce diagnostic odysseys. For instance, identifying pathogenic variants in genes allows early initiation of targeted therapies, significantly improving outcomes.

**Conclusions:**

Despite its transformative potential, challenges remain, including cost, data interpretation, and equitable access. Addressing these barriers through investments in infrastructure, training, and policy frameworks will be crucial for broader implementation. This review underscores the critical role of rWGS in neonatal care and highlights its promise as a cornerstone of precision medicine, paving the way for improved diagnostic accuracy and patient outcomes in metabolic diseases.

## 1 Introduction

Metabolic diseases encompass a broad spectrum of disorders characterized by disruptions in the biochemical pathways essential for energy production, growth, and overall homeostasis (1). These disorders are often caused by genetic mutations affecting enzymes, transport proteins, or other key regulators of metabolic processes. Examples include lysosomal storage diseases, urea cycle disorders, fatty acid oxidation defects, and mitochondrial diseases, among others (2). Collectively, metabolic diseases represent a significant clinical burden, with an estimated prevalence of 1 in 800 live births for inherited metabolic disorders alone, and higher rates when considering acquired metabolic syndromes. Recent international efforts in the International Classification of Inherited Metabolic Disorders (ICIMD) identified nearly 1,500 distinct conditions as inherited metabolic disorders, reflecting the expansion of recognized disorders as diagnostic technologies rapidly advanced (3). Many of these diseases manifest in infancy or early childhood and, if untreated, can lead to life-threatening crises, permanent disability, or early mortality (4).

The economic and societal costs of metabolic diseases are profound. Prolonged diagnostic odysseys, commonly experienced by patients with rare metabolic disorders, contribute to delayed or missed opportunities for timely intervention. Such delays often lead to irreversible complications, exacerbating healthcare costs and impacting quality of life (5). Despite advances in diagnostic modalities, many cases remain unresolved due to the genetic heterogeneity of these disorders and the limitations of traditional diagnostic approaches. Addressing these challenges demands innovative tools capable of rapid and comprehensive genetic assessment, making genomic technologies indispensable in modern medicine (6, 7). Recent findings by Stranneheim & Wedell highlight how whole-exome and whole-genome sequencing have revolutionized diagnostic practices, especially for monogenic diseases. Their review underscored that traditional diagnostic workflows often overlook complex or overlapping phenotypes, leading to delayed or missed diagnoses. These limitations are exacerbated in metabolic diseases due to their rapid onset and genetic variability, further justifying the push toward genome-wide approaches such as rWGS (7).

The advent of genomics has revolutionized our understanding of metabolic diseases, shedding light on their molecular underpinnings. Historically, diagnostic approaches for metabolic conditions relied heavily on biochemical assays, tandem mass spectrometry, and enzyme activity tests. While these methods remain valuable, they often fail to identify the underlying genetic cause, particularly in atypical cases or disorders without known biomarkers (Table 1) (8, 9). Traditional diagnostic methods, including biochemical assays and single-gene testing, are often time-consuming and may not provide definitive results. Indeed, recent systematic evidence shows that up to 85% of metabolic diseases ultimately require next-generation sequencing (exome or genome sequencing) for a definitive diagnosis (10).

**Table 1:** Comparison of diagnostic methods for metabolic diseases.

Diagnostic method	Average turnaround time	Diagnostic yield (%)x	Strengths	Limitations
Biochemical assays	1–7 days	30%–40%	Inexpensive; well-established	Limited to known biomarkers; lacks genetic insights
Tandem mass spectrometry	1–3 days	40%–50%	Effective for metabolic markers	Cannot detect genetic causes or novel biomarkers
Enzyme activity tests	2–5 days	30%–50%	Direct measurement of enzyme deficiencies	Invasive (e.g., biopsies); limited specificity
Targeted gene panels	2–4 weeks	20%–30%	Focused genetic testing for known disorders	Miss novel or atypical variants
Rapid WGS (rWGS)	24–72 h	50%–70%	Comprehensive genetic assessment; fast results	High cost; bioinformatics expertise required

Genomic technologies, such as whole-exome sequencing (WES) and whole-genome sequencing (WGS), have bridged this gap by enabling direct interrogation of the entire coding and non-coding genome. These approaches have uncovered novel disease-associated genes, expanded our knowledge of genotype-phenotype correlations, and provided a basis for precision medicine interventions (11, 12). For instance, discoveries of pathogenic variants in genes such as GLDC (glycine encephalopathy), MMUT (methylmalonic aciduria), and GAA (Pompe disease) have not only elucidated disease mechanisms but also informed therapeutic strategies, including enzyme replacement therapies and small-molecule treatments (13).

Despite these advances, traditional WGS has been hindered by logistical challenges, including long turnaround times, high costs, and complex data interpretation (14). In a recent qualitative study, Friedrich et al. interviewed clinicians during the early implementation of a national genomic medicine service in the UK. They emphasized the importance of real-time support systems and training to ensure that rWGS can be effectively interpreted and translated into clinical practice. Their findings suggest that genomic technologies, despite their promise, require systemic readiness and cultural adaptation in hospital environments (14). These barriers are particularly consequential in metabolic diseases, where timely diagnosis can mean the difference between survival and death. It is within this context that rapid whole genome sequencing (rWGS) has emerged as a transformative tool, offering unprecedented speed and diagnostic efficiency (15, 16).

**Figure 1:**
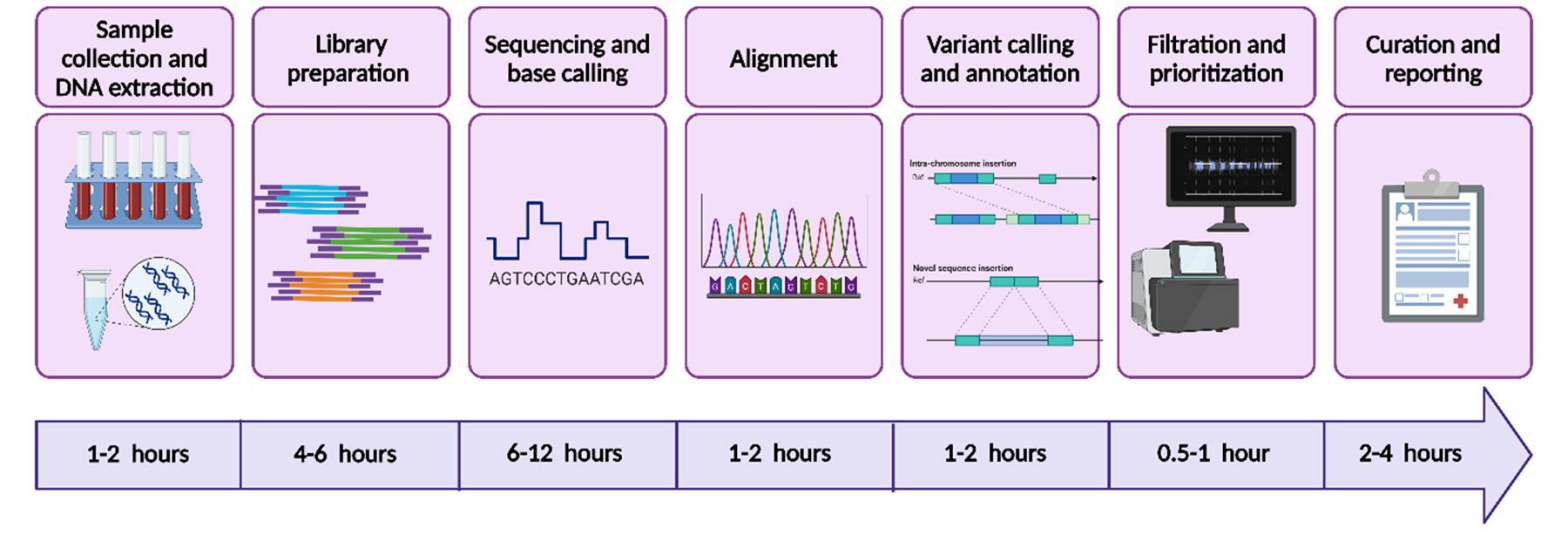
Overall step-by-step workflow of the rapid whole sequencing (rWGS) along with a timeline at each step. Created in https://BioRender.com.

Rapid whole genome sequencing (rWGS) represents a pivotal advancement in genomic medicine, particularly in the diagnosis and management of metabolic diseases. Unlike traditional WGS, which may take weeks to yield results, rWGS enables the comprehensive analysis of an individual's entire genome within 24–72 h (Figure 1) (16). This acceleration is achieved through a combination of optimized sequencing workflows, high-throughput platforms, and advanced computational pipelines that prioritize clinically relevant variants. Such rapidity is especially critical for patients in acute settings, including neonates in intensive care units (NICUs), where diagnostic uncertainty can have life-threatening consequences (17).

Technological advancements have been instrumental in enabling rWGS. For instance, the latest generation of sequencing platforms, such as Illumina NovaSeq and Oxford Nanopore Technologies' PromethION, offer unparalleled read depth, accuracy, and speed (18). Concurrently, bioinformatics tools leveraging artificial intelligence and machine learning (19) algorithms have streamlined variant annotation, prioritization, and interpretation. These innovations have reduced the time and expertise required for data analysis, thereby enhancing the clinical utility of rWGS (20). As detailed by Wang et al., Oxford Nanopore's PromethION has gained traction not only for its real-time sequencing capabilities but also for its capacity to detect base modifications and large-scale structural variants, features often missed by short-read technologies. Their study demonstrated how nanopore platforms can complement traditional short-read methods, particularly in diagnosing metabolic conditions with complex variant types or repetitive genomic regions (20).

The clinical impact of rWGS in metabolic diseases is underscored by its high diagnostic yield, which often exceeds 50% in critically ill populations. Studies have demonstrated that rWGS not only accelerates diagnosis but also alters clinical management in a significant proportion of cases, facilitating early interventions that improve outcomes (21). For example, identifying a pathogenic variant in FAH (fumarylacetoacetate hydrolase) can prompt early initiation of nitisinone therapy in tyrosinemia type 1, preventing hepatic and renal complications (22). Similarly, rWGS has been instrumental in diagnosing rare mitochondrial disorders, enabling targeted nutritional and pharmacological interventions (23).

Beyond individual patient care, rWGS has far-reaching implications for public health and research. Its integration into newborn screening programs could enable the early detection of metabolic diseases at a population level, ensuring timely treatment and reducing the long-term burden of disease (24). Additionally, the data generated through rWGS has the potential to uncover novel disease mechanisms, identify therapeutic targets, and refine our understanding of disease variability (25).

The diagnostic odyssey faced by many patients with metabolic diseases highlights the limitations of existing diagnostic paradigms. Traditional approaches often involve sequential testing, with each step requiring time and resources that delay definitive diagnosis. In many cases, patients undergo numerous invasive procedures, including muscle biopsies and lumbar punctures, before a genetic cause is identified. This process not only imposes physical and emotional burdens on patients and families but also delays the initiation of life-saving therapies (26).

One of the primary challenges lies in the genetic heterogeneity of metabolic diseases. A single clinical phenotype, such as hypoglycemia or developmental delay, can result from mutations in numerous genes, making targeted testing inefficient. Furthermore, overlapping biochemical profiles often complicate differential diagnosis. For example, disorders of energy metabolism, such as mitochondrial diseases and fatty acid oxidation defects, can present with similar clinical features but require distinct management strategies. The ability of rWGS to provide a comprehensive genetic assessment in a single test addresses these limitations, offering a unified diagnostic approach for diverse presentations (27)

Another critical challenge is the early identification of metabolic diseases in asymptomatic individuals, particularly in the neonatal period. While tandem mass spectrometry-based newborn screening has significantly improved early detection, it is limited to conditions with known biomarkers. Many metabolic diseases remain undetectable using current screening panels, leading to missed diagnoses. rWGS has the potential to overcome this limitation by enabling genetic screening for a broader range of conditions, including those without established biomarkers. This capability is particularly relevant in cases of atypical or novel presentations, where traditional methods are unlikely to provide answers (28).

The integration of rWGS into clinical workflows represents a paradigm shift in the diagnosis and management of metabolic diseases. By providing rapid and comprehensive genetic insights, rWGS enables precision medicine approaches that were previously unattainable. Its potential to reduce diagnostic delays, avoid unnecessary procedures, and inform targeted therapies underscores its transformative impact on patient care (29).

Moreover, rWGS aligns with broader trends in healthcare, including the shift toward personalized and predictive medicine. As the cost of sequencing continues to decline, the accessibility of rWGS is likely to increase, making it feasible for routine use in diverse healthcare settings. The inclusion of rWGS in insurance coverage and national health programs will further drive its adoption, democratizing access to genomic medicine.

In this review, we explore the current state of rWGS in the context of metabolic diseases, focusing on its applications, impact, and future directions. We begin by examining the technical foundations of rWGS, including advancements in sequencing technologies and bioinformatics. We then discuss its clinical applications, highlighting real-world case studies and evidence of improved outcomes. Finally, we address the challenges and limitations of rWGS and outline emerging trends that are poised to shape its future. By providing a comprehensive overview of this rapidly evolving field, we aim to underscore the critical role of rWGS in advancing the diagnosis and treatment of metabolic diseases.

## 2 Technical foundations of rapid whole genome sequencing (rWGS)

rWGS has emerged as a transformative tool in the diagnosis of genetic diseases, particularly metabolic disorders, where time-sensitive interventions can save lives. The technological advancements underlying rWGS enable comprehensive genomic analysis at unprecedented speed, accuracy, and scale (16). This section examines the technical foundations of rWGS, focusing on sequencing platforms, computational pipelines, speed vs. accuracy trade-offs, and the economic and global accessibility of this technology.

### 2.1 Advances in sequencing technology

The success of rWGS is fundamentally underpinned by advances in sequencing technologies. Next-generation sequencing platforms, which have revolutionized genomic medicine, continue to evolve in terms of throughput, accuracy, and cost-efficiency. The three dominant platforms in clinical sequencing, Illumina, Pacific Biosciences (PacBio), and Oxford Nanopore Technologies, each contribute unique strengths to the application of rWGS (Table 2). Recently, pilot projects have also been initiated to evaluate the feasibility of rapid long-read genome sequencing in clinical settings, further expanding the diagnostic capabilities beyond conventional short-read methods (30). While short-read sequencing has proven invaluable for diagnosing a wide range of metabolic disorders, its technical limitations include reduced sensitivity for detecting structural variants, repeat expansions, and complex genomic rearrangements. Long-read sequencing technologies are poised to address these gaps by providing improved resolution across challenging genomic regions.

**Table 2:** Technical comparison of sequencing platforms.

Platform	Read type	Turnaround time (approx.)	Strengths	Weaknesses
llumina (NovaSeq)	Short Reads	1–3 days	High accuracy; cost-effective	Limited for structural variants
PacBio (HiFi)	Long Reads	72 h	Resolves complex regions and structural variants	Higher cost; slower turnaround
Oxford Nanopore	Ultra-Long Reads	6–12 h	Real-time data; portable	Historically lower accuracy

#### 2.1.1 Illumina: high-throughput and established accuracy

Illumina's short-read sequencing platforms, such as the NovaSeq 6000, dominate the clinical sequencing landscape due to their unparalleled accuracy and high throughput. Illumina sequencing employs a sequencing-by-synthesis (SBS) technology, which generates reads of up to 300 base pairs (bp) with high fidelity. The platform is particularly well-suited for detecting small variants such as single-nucleotide polymorphisms (SNPs) and small insertions or deletions (indels). In the context of rWGS, Illumina has optimized workflows to minimize sequencing time. Advances in library preparation, cluster generation, and real-time data processing have enabled whole-genome sequencing results within 24–48 h. For example, the TruSight Rapid Capture kit allows for library preparation and enrichment within a few hours, significantly reducing total turnaround time. Coupled with its robust bioinformatics ecosystem, Illumina remains the gold standard for rWGS in clinical settings (18).

#### 2.1.2 Pacific biosciences (PacBio): long-read accuracy for complex regions

PacBio's single-molecule real-time (SMRT) sequencing technology offers a complementary approach to Illumina's short-read platforms by providing long-read sequencing with read lengths exceeding 10,000 bp. This capability makes PacBio particularly valuable for resolving complex genomic regions such as repetitive sequences, structural variants (SVs), and regions of high GC content, which are often implicated in metabolic diseases. The introduction of PacBio's HiFi reads, which combine long-read lengths with high accuracy (>99%), has further enhanced the platform's clinical utility. Although PacBio sequencing is slower and more expensive than Illumina, it excels in identifying pathogenic variants that may be missed by short-read platforms, underscoring its importance in rWGS workflows where comprehensive variant detection is critical (31).

#### 2.1.3 Oxford nanopore technologies: real-time and portability

Oxford Nanopore Technologies (32) has pioneered real-time sequencing with its nanopore-based platforms, such as the PromethION and MinION. ONT sequencing involves passing DNA molecules through protein nanopores, allowing for the direct detection of nucleotide sequences in real time. This approach enables ultra-long reads (>100,000 bp) and can sequence modified bases, such as methylation, directly. ONT's rapid library preparation protocols and real-time data generation make it particularly well-suited for time-sensitive clinical applications. In emergency settings, ONT has demonstrated the ability to generate actionable genomic data within 6–12 h. However, the platform's accuracy, historically lower than Illumina or PacBio, has improved with algorithmic advancements such as base-calling using neural networks (e.g., Guppy software) (33).

### 2.2 Computational pipelines and AI in variant detection and prioritization

The utility of rWGS extends beyond sequencing speed; rapid and accurate interpretation of the vast amounts of genomic data generated is equally critical. Computational pipelines have been optimized to facilitate the identification, annotation, and prioritization of clinically relevant variants within a compressed timeframe (34).

Modern rWGS pipelines rely on advanced algorithms for aligning sequencing reads to a reference genome, identifying variants, and filtering noise. Tools such as BWA-MEM and Minimap2 are widely used for alignment, while variant callers like GATK and DeepVariant achieve high sensitivity and specificity for SNPs and indels. Annotation tools, including ANNOVAR, VEP (Variant Effect Predictor), and ClinVar databases, provide functional and clinical context to identified variants (35).

Artificial intelligence and machine learning (19) have revolutionized the interpretation of genomic data. AI-driven tools, such as Emedgene, Fabric Genomics, and DeepGestalt, use phenotype-based prioritization to rank variants based on their clinical relevance. By integrating patient-specific data, including phenotypic descriptors encoded using systems like Human Phenotype Ontology (HPO), these tools can highlight the most probable pathogenic variants, dramatically reducing the time required for manual curation. Furthermore, neural networks and deep learning models are increasingly applied to structural variant detection, copy number variant (CNV) analysis, and functional prediction. These advancements ensure that rWGS pipelines not only operate quickly but also maintain high diagnostic accuracy (36).

### 2.3 Speed vs. accuracy trade-offs

One of the defining features of rWGS is its ability to balance speed and accuracy, particularly in clinical scenarios requiring urgent diagnoses. However, achieving this balance involves trade-offs that depend on the clinical context and the capabilities of sequencing platforms (37).

Traditional WGS workflows often require weeks to complete sequencing, analysis, and reporting (38). In contrast, rWGS workflows, optimized for critically ill patients, aim for turnaround times as short as 24–72 h (39). Key factors enabling this rapid pace include 1) accelerated library preparation protocols, 2) high-throughput sequencing platforms, 3) streamlined computational pipelines for variant calling and prioritization. Studies have demonstrated that rWGS can achieve diagnostic yields of 50%–60% within these timelines, providing actionable results that influence clinical management (40).

While speed is a priority in rWGS, it must not come at the expense of diagnostic accuracy. Short-read platforms like Illumina, while fast, may miss structural variants and variants in complex regions. Long-read platforms such as PacBio and ONT, while slower, provide deeper insights into these regions. Hybrid approaches that combine short- and long-read data are increasingly being explored to maximize both speed and diagnostic yield (31).

Another trade-off involves read depth. Higher coverage improves variant detection but increases sequencing time and cost. Clinical applications of rWGS often aim for coverage of 30× or higher, balancing diagnostic sensitivity with workflow efficiency.

### 2.4 Cost and accessibility

Despite its transformative potential, the widespread implementation of rWGS faces economic and logistical barriers. These challenges vary across healthcare systems and regions, influencing the accessibility of this technology.

The cost of WGS has declined dramatically over the past decade, with sequencing costs approaching $200 per genome in research settings. However, the total cost of rWGS in clinical practice, typically ranging from $5,000 to $10,000 per patient, remains a barrier to adoption. This cost includes sequencing, bioinformatics analysis, and interpretation by clinical geneticists.

Efforts to reduce costs are ongoing, with innovations in library preparation, sequencing efficiency, and automation driving down expenses. Additionally, health economic studies have shown that rWGS can be cost-effective in the long term by reducing unnecessary diagnostic tests, shortening hospital stays, and enabling timely interventions.

While high-income countries increasingly adopt rWGS in clinical care, resource-limited settings face significant barriers. These include limited access to sequencing infrastructure, a shortage of trained bioinformaticians and clinical geneticists, and high upfront costs for equipment and consumables (41).

Efforts to address these disparities include initiatives such as the H3Africa consortium, which aims to expand genomic medicine in Africa, and the development of portable sequencing devices like ONT's MinION, which require minimal infrastructure.

## 3 Applications of rapid whole genome sequencing in metabolic diseases

The utility of rapid whole genome sequencing (rWGS) in metabolic diseases lies in its ability to provide timely and comprehensive genetic diagnoses, which are critical for conditions where early intervention can drastically alter outcomes. Next-generation sequencing provides comprehensive coverage across diverse metabolic pathways and is critical for establishing molecular diagnoses. This is underscored by recent findings that approximately 85% of metabolic disease cases require exome or genome sequencing for a definitive diagnosis (10).

This section explores the diverse applications of rWGS in clinical practice, including neonatal and pediatric care, resolution of diagnostic odysseys in rare diseases, precision medicine, and prenatal screening. Each of these areas highlights the transformative role of rWGS in improving patient outcomes and advancing personalized medicine.

### 3.1 Neonatal and pediatric applications

Metabolic diseases frequently present in neonates and children, often manifesting as life-threatening crises that require immediate diagnosis and intervention (42). The traditional diagnostic approach for critically ill neonates, relying on sequential biochemical assays and imaging studies, can be time-consuming and insufficient for rare or atypical conditions. rWGS has revolutionized this paradigm, offering a single test that rapidly identifies genetic causes, thus enabling timely and targeted interventions (43).

NICUs are among the most active settings for rWGS implementation. Critically ill neonates often present with non-specific symptoms such as seizures, hypotonia, or failure to thrive, making the differential diagnosis extensive and challenging. rWGS allows clinicians to bypass lengthy diagnostic odysseys by providing a genetic diagnosis within 24–72 h. This speed is crucial for initiating disease-specific treatments, particularly in time-sensitive metabolic disorders like urea cycle defects and maple syrup urine disease (MSUD), where delays can result in irreversible neurological damage or death (44). Sanford et al. (45) reported on a cohort of children in the PICU who underwent rWGS, showing that nearly half received a genetic diagnosis, and in over one-third of cases, clinical management was altered. These changes included initiation of enzyme therapies, surgical interventions, and changes in medication, demonstrating that rWGS is not merely diagnostic but often therapeutic in its implications. This is particularly impactful in metabolic diseases, where early identification can be lifesaving (44).

Studies have consistently demonstrated the high diagnostic yield of rWGS in NICUs. For example, a landmark study by Kingsmore et al. reported a diagnostic yield of 57% in critically ill infants, with changes in clinical management implemented in 52% of cases following rWGS results (46). Similarly, in a multicenter study, rapid sequencing led to actionable findings in over half of the cases, significantly improving survival rates and reducing hospital stays (16).

Another case involved a neonate with severe hypotonia and respiratory distress, initially suspected to have a neuromuscular disorder. rWGS revealed a pathogenic variant in GAA, confirming Pompe disease. Early enzyme replacement therapy was initiated, significantly improving the child's prognosis (4). These cases underscore the unique value of rWGS in delivering rapid, definitive diagnoses that directly inform lifesaving treatments (45).

### 3.2 Undiagnosed rare diseases

For patients with rare metabolic diseases, diagnostic odysseys often span years, involving multiple inconclusive tests and significant emotional and financial burdens. rWGS has emerged as a powerful tool for resolving these odysseys by uncovering genetic causes that traditional methods may miss (16).

Rare metabolic disorders, such as mitochondrial diseases, lysosomal storage disorders, and peroxisomal biogenesis defects, often present with overlapping clinical and biochemical features. rWGS enables comprehensive genetic assessment in a single test, eliminating the need for sequential targeted testing. A recent meta-analyses reported that rWGS successfully resolved diagnostic odysseys in approximately 40%–60% of cases, with higher yields in cases involving neurological or metabolic phenotypes (47, 48).

Mitochondrial diseases, which involve defects in oxidative phosphorylation, are among the most challenging to diagnose due to their genetic heterogeneity. rWGS has been instrumental in identifying mutations in nuclear and mitochondrial DNA that contribute to these disorders. For example, variants in POLG and SUCLA2 have been linked to mitochondrial depletion syndromes, guiding decisions on dietary and cofactor supplementation (49).

Lysosomal storage diseases, such as Gaucher disease and Fabry disease, also benefit from rWGS. In one study, rWGS identified a previously undiagnosed case of Niemann-Pick disease type C, allowing for targeted therapy with miglustat to be initiated. These findings emphasize the potential of rWGS to illuminate rare, actionable diagnoses in previously undiagnosed patients (50).

### 3.3 Pharmacogenomics and precision medicine

rWGS not only facilitates diagnosis but also plays a critical role in pharmacogenomics, enabling personalized therapeutic strategies based on genetic insights. By identifying actionable variants, rWGS empowers clinicians to tailor treatments that maximize efficacy and minimize adverse effects.

A significant advantage of rWGS lies in its ability to detect pharmacogenomic markers that influence drug metabolism and response. For instance, rWGS can identify mutations in genes such as CYP2D6 and DPYD, which impact the metabolism of commonly used medications like opioids and chemotherapeutic agents. This information is particularly relevant in metabolic diseases, where certain drugs may exacerbate symptoms or lead to toxic accumulation (51).

Precision medicine approaches are especially valuable in metabolic diseases with specific therapeutic targets. For example, patients with phenylketonuria (PKU) who carry mutations responsive to tetrahydrobiopterin (BH4) can benefit from cofactor therapy identified through genetic analysis (52). Similarly, rWGS can identify patients with GCDH mutations (glutaric aciduria type 1) who require carnitine supplementation and lysine-restricted diets to prevent metabolic crises (53).

Gene therapy is another area where rWGS is poised to make a substantial impact. By pinpointing the precise genetic cause of disease, rWGS can guide the development and application of gene-editing tools such as CRISPR-Cas9 or using AAV vectors. Ongoing clinical trials in metabolic diseases like Aromatic L-Amino Acid Decarboxylase Deficiency illustrate the potential of these approaches (54).

### 3.4 Prenatal and carrier screening

rWGS has significant implications for prenatal care and family planning, offering insights into the genetic basis of inherited metabolic diseases before symptoms manifest. This capability enables early interventions and informed decision-making for prospective parents.

While traditional NIPT focuses on detecting chromosomal aneuploidies, advances in sequencing technology now allow for the detection of single-gene disorders and pathogenic variants associated with metabolic diseases. By sequencing cell-free fetal DNA (cffDNA) from maternal plasma, rWGS can identify variants associated with conditions such as congenital adrenal hyperplasia and inborn errors of metabolism. This approach expands the diagnostic scope of prenatal testing, providing valuable information about fetal health without invasive procedures (55).

Carrier screening using rWGS enables couples to assess their risk of passing on metabolic disorders to their offspring. This is particularly relevant for autosomal recessive conditions, where both parents must carry a pathogenic variant. For example, screening for spinal muscular atrophy, Tay-Sachs disease, and cystic fibrosis has been integrated into routine care in many regions (56), allowing for preimplantation genetic diagnosis and other reproductive options (57).

In cases where rWGS identifies a fetal genetic disorder, early interventions can be planned. For instance, pregnancies affected by metabolic conditions such as methylmalonic acidemia may benefit from planned deliveries in specialized centers equipped to provide immediate metabolic management. These advancements underscore the role of rWGS in promoting proactive and personalized prenatal care (58).

## 4 Key findings and impact on patient outcomes

The implementation of rWGS in clinical practice has led to significant improvements in diagnostic accuracy, time to diagnosis, and patient outcomes, particularly in the context of metabolic diseases. This section explores the evidence supporting the efficacy of rWGS compared to traditional diagnostic approaches, provides transformative case examples, and discusses the real-world integration of rWGS into multidisciplinary care pathways.

### 4.1 Diagnostic yield and efficacy

One of the most compelling arguments for the adoption of rWGS is its superior diagnostic yield compared to standard diagnostic methods. Traditional diagnostic approaches for genetic diseases often involve a sequential battery of tests, including biochemical assays, imaging studies, and targeted genetic panels, which are time-consuming and can result in missed or delayed diagnoses (9, 59). In contrast, rWGS offers a single, comprehensive test capable of identifying a wide range of genetic variants.

Several studies have demonstrated the enhanced diagnostic yield of rWGS in both critically ill and outpatient populations. For example, a study by Farnaes et al. reported a diagnostic yield of 43% in critically ill infants undergoing rWGS, compared to 10% for standard diagnostic methods (60). Similarly, in a multicenter study involving 184 neonates with suspected genetic disorders, it was found that rWGS achieved a diagnostic yield of 57%, with actionable findings in 52% of cases that directly influenced clinical management (61). Notably, Farnaes et al. demonstrated that rWGS reduced hospitalization time and overall costs by providing rapid diagnoses in neonates with suspected metabolic disorders. Their data revealed that earlier treatment interventions, guided by genomic findings, avoided extensive and invasive testing, supporting the long-term cost-efficiency of rWGS integration in NICU settings (60).

These findings are further supported by several meta-analyses of rWGS studies, which report an average diagnostic yield of 40%–60% in metabolic and neurological disorders, compared to 10%–20% for traditional methods (47, 48). The ability of rWGS to detect single-nucleotide variants (SNVs), copy number variants (CNVs), and structural variants in a single test underpins its diagnostic superiority.

Rapid turnaround times are critical in metabolic diseases, where delayed diagnosis can result in irreversible organ damage or death. Traditional diagnostic workflows often require weeks or months to yield definitive results, whereas rWGS can provide actionable findings within 24–72 h. Studies have shown that the use of rWGS reduces the average time to diagnosis by 77%–90% compared to standard approaches. This accelerated timeline is particularly impactful in NICUs, where time-sensitive interventions are frequently needed (16).

The diagnostic efficiency of rWGS translates directly into improved patient outcomes. Early identification of metabolic diseases allows for the timely initiation of disease-specific treatments, such as dietary modifications, enzyme replacement therapies, or ammonia scavenger therapies. A 2022 study by Kobayashi et al. found that rWGS reduced the length of hospital stays and healthcare costs while significantly improving survival rates in critically ill infants with metabolic crises (62).

### 4.2 Real-world implementation

Beyond aggregate data, individual cases illustrate the profound clinical impact of rWGS in resolving diagnostic dilemmas and guiding life-saving interventions (50, 63, 64). These transformative cases highlight the clinical utility of rWGS in delivering definitive diagnoses and guiding precise therapeutic interventions.

The successful integration of rWGS into routine clinical care requires coordinated efforts by multidisciplinary teams and institutional support to overcome logistical, technical, and financial challenges. The interpretation and application of rWGS results demand expertise from various disciplines, including clinical geneticists, bioinformaticians, metabolic specialists, and genetic counselors. Multidisciplinary teams play a critical role in interpreting variants of uncertain significance (VUS) providing phenotypic data to prioritize pathogenic variants, and counseling patients and families about the implications of genetic findings (65). For example, rWGS findings of a VUS in a gene associated with metabolic myopathy may require functional studies or additional biochemical testing to confirm pathogenicity. Collaboration between geneticists and metabolic specialists is essential to translate such findings into actionable clinical decisions.

Real-world studies have demonstrated that the integration of rWGS into clinical workflows improves patient care pathways and decision-making. In a study involving critically ill neonates, the use of rWGS resulted in changes to clinical management, including the initiation of targeted therapies, discontinuation of unnecessary treatments, and informed decisions about palliative care. Additionally, rWGS has facilitated earlier transitions to specialized care centers for patients with complex metabolic conditions. For instance, patients diagnosed with mitochondrial disorders through rWGS are often referred to centers of excellence for tailored therapies and experimental trials (66). A systematic review by Ma et al. found that genomic multidisciplinary teams not only improved diagnostic accuracy but also increased physician confidence in acting on genetic findings. This highlights the necessity of team-based genomic medicine, especially for complex disorders like metabolic diseases where coordinated interpretation of biochemical, clinical, and genomic data is crucial for decision-making (65).

Despite its benefits, the real-world implementation of rWGS faces several challenges. The high cost of rWGS remains a barrier to widespread adoption. While health economic studies have shown its cost-effectiveness in reducing diagnostic delays and hospitalizations, insurance coverage for rWGS varies widely across countries and healthcare systems (62). The integration of rWGS requires significant investment in sequencing infrastructure, computational resources, and trained personnel. The interpretation of genomic data remains complex, with challenges in assessing the clinical significance of VUS and incidental findings. False positives are a notable concern, particularly in the early phases of clinical implementation. A study by Narravula et al. emphasized that VUS often appear in newborn screening programs and can lead to overdiagnosis or unnecessary anxiety. They recommend structured reanalysis and functional validation pipelines to reduce clinical misinterpretation of such results, a process increasingly supported by AI-based reclassification tools (67). Ongoing efforts to expand variant databases and develop AI-driven tools aim to address these issues.

## 5 Challenges and limitations of rapid whole genome sequencing

Despite its transformative potential, rapid whole genome sequencing (rWGS) faces several challenges that limit its widespread adoption and integration into routine clinical practice. These challenges span bioinformatics, ethics, resources, and clinical accuracy. Addressing these issues is critical for maximizing the utility of rWGS in diagnosing and managing metabolic diseases. This section outlines the primary obstacles and discusses strategies for overcoming them.

### 5.1 Bioinformatics and data interpretation

The interpretation of rWGS data remains one of the most significant hurdles in clinical genomics. The sequencing process generates vast amounts of data, requiring sophisticated bioinformatics tools and expert interpretation to identify clinically relevant variants.

One of the key challenges in interpreting rWGS results is the identification of VUS. These are genetic variants whose pathogenicity cannot be conclusively determined based on current knowledge. VUS are particularly problematic in rare metabolic diseases, where genotype-phenotype correlations may be poorly defined. In some studies, VUS account for up to 20%–30% of identified variants in rWGS analyses (67). The clinical management of patients with VUS is complicated, as these findings can create diagnostic uncertainty and raise ethical questions about disclosure. For example, in cases where a VUS is identified in a gene associated with a metabolic disorder, clinicians must decide whether to initiate treatment based on incomplete evidence or wait for further data, potentially delaying care.

Robust genomic databases and annotation tools are essential for accurate variant interpretation. However, current databases, such as ClinVar, HGMD (Human Gene Mutation Database), and gnomAD, have limitations in terms of completeness and population diversity. Many variants identified in non-European populations are underrepresented, leading to potential biases in interpretation (68). Improving the utility of these databases requires expanding population-specific variant data to increase representativeness, integrating functional studies and in silico prediction tools to refine variant classification, incorporating real-world clinical outcomes to enhance genotype-phenotype correlations. Advances in artificial intelligence and machine learning (19) are also being applied to improve annotation and prioritization of variants. AI-driven tools, such as DeepVariant and Exomiser, are helping to streamline data analysis and reduce the burden of manual interpretation (69).

### 5.2 Ethical and legal considerations

The implementation of rWGS raises complex ethical and legal questions related to informed consent, incidental findings, and patient privacy. Obtaining informed consent for rWGS is more complex than for traditional diagnostic tests due to the comprehensive nature of genomic data. Patients must understand that rWGS can reveal information beyond the primary indication for testing, including secondary findings or susceptibility to unrelated conditions. Communicating these possibilities effectively is challenging, particularly in time-sensitive situations such as NICUs (70). Emerging frameworks for dynamic consent, which allow patients to update their preferences over time, may help address these challenges. Additionally, clear communication strategies and decision aids are needed to support families in making informed choices.

The potential for incidental findings, unanticipated genetic results unrelated to the primary reason for testing, poses ethical dilemmas. For example, identifying a BRCA1 mutation in a child undergoing rWGS for a metabolic disorder raises questions about whether to disclose this information, especially when it has implications for adult-onset conditions (71). The American College of Medical Genetics and Genomics (ACMG) recommends reporting specific actionable incidental findings, but the approach to managing non-actionable or uncertain findings remains debated (72). Developing standardized guidelines for incidental findings disclosure is critical for ensuring consistency and minimizing harm.

The sensitive nature of genomic data requires robust safeguards to protect patient privacy. The storage and sharing of rWGS data increase the risk of breaches, unauthorized access, and misuse, raising concerns about discrimination and stigmatization. Compliance with regulations such as the General Data Protection Regulation (GDPR) in Europe and the Health Insurance Portability and Accountability Act (HIPAA) in the U.S. is essential but can be logistically challenging for healthcare institutions (73). Data encryption, anonymization, and controlled access protocols are vital for ensuring security. Moreover, fostering public trust in genomic medicine will require transparent policies and accountability mechanisms.

### 5.3 Resource barriers

The resource-intensive nature of rWGS limits its accessibility, particularly in low- and middle-income countries (LMICs). These barriers include infrastructure, workforce training, and financial constraints.

Performing rWGS requires advanced sequencing platforms, computational resources, and laboratory infrastructure. Many healthcare facilities, particularly in LMICs, lack the resources to establish and maintain these systems. Portable sequencing technologies, such as Oxford Nanopore's MinION, may provide cost-effective alternatives, but their accuracy and throughput currently lag behind high-throughput platforms like Illumina's NovaSeq. The successful implementation of rWGS depends on a trained workforce, including geneticists, bioinformaticians, and genetic counselors (74). A global shortage of skilled professionals remains a significant barrier to scaling rWGS. Addressing this gap will require expanding training programs in genomic medicine, developing user-friendly bioinformatics tools to reduce reliance on specialized expertise, promoting international collaborations to share expertise and resources.

Global disparities in access to rWGS reflect broader inequities in healthcare. While high-income countries increasingly integrate rWGS into clinical care, many LMICs struggle to provide even basic diagnostic services for metabolic diseases. Initiatives such as the H3Africa consortium aim to address these inequities by building genomic capacity in underserved regions (75). Scaling rWGS globally will require innovative funding models, such as public-private partnerships, and advocacy for the inclusion of genomic medicine in national health policies.

### 5.4 False positives and negatives in clinical use

The clinical accuracy of rWGS depends on its ability to minimize false-positive and false-negative results, which can have serious consequences for patient care.

False-positive results occur when variants are incorrectly classified as pathogenic. These errors can lead to unnecessary treatments, anxiety, and additional testing. For example, misclassification of benign variants in genes associated with metabolic disorders may result in inappropriate dietary or pharmacological interventions (76). To mitigate false positives: 1) Variant classification systems, such as the ACMG guidelines, should be rigorously applied. 2) Functional validation studies and segregation analyses should be performed when possible. 3) Clinicians should consider the patient's phenotype and biochemical findings when interpreting rWGS results.

False negatives, where pathogenic variants are missed, remain a challenge, particularly for certain variant types and genomic regions. Short-read sequencing platforms, such as Illumina, may fail to detect large structural variants, repetitive sequences, or regions of high GC content. Long-read sequencing platforms, such as PacBio and Oxford Nanopore, can address some of these limitations but are not yet widely used in clinical settings (77). Improving bioinformatics pipelines, incorporating hybrid sequencing approaches, and updating reference genomes are essential for reducing false negatives and ensuring comprehensive variant detection.

It is important to emphasize that biochemical testing and rWGS are complementary rather than mutually exclusive approaches. For example, classical disorders such as phenylketonuria, urea cycle defects, and methylmalonic acidemia are rapidly and reliably confirmed by plasma or urine metabolite assays, whereas rWGS may yield uncertain variant calls or require additional interpretation time. Conversely, rWGS is particularly effective and required for conditions with heterogeneous or subtle biochemical phenotypes, such as molybdenum cofactor deficiency, CblX remethylation disorder, or several lysosomal storage disorders, such as Niemann-Pick disease type C, where biochemical markers may be absent, nonspecific, or inconclusive. These examples demonstrate that optimal patient care requires integrating rapid genomic sequencing with targeted biochemical assays. Therefore, using each method's strengths might overcome the other's limitations.

## 6 Emerging trends and future directions in rWGS

rWGS continues to evolve, its integration with emerging technologies and expanding applications holds significant promise for advancing the diagnosis and treatment of metabolic diseases. This section explores key trends and future directions, including the convergence of rWGS with other omics approaches, the role of artificial intelligence (6) in enhancing clinical interpretation, advancements in portable sequencing technologies, and the increasing role of rWGS in personalized therapies such as gene editing.

### 6.1 Integration with other omics

One of the most exciting frontiers in rWGS lies in its integration with other omics approaches, including epigenomics, metabolomics, and transcriptomics. Together, these technologies offer a more comprehensive understanding of the molecular mechanisms underlying metabolic diseases and enable a systems biology approach to precision medicine (78).

Epigenetic modifications, such as DNA methylation, histone modifications, and non-coding RNAs, play a critical role in regulating gene expression and metabolic pathways. Aberrant epigenetic changes have been implicated in several metabolic disorders, including mitochondrial dysfunction and lysosomal storage diseases. Integrating epigenomic profiling with rWGS can help elucidate the functional consequences of genetic variants and identify secondary regulatory mechanisms that contribute to disease progression (79, 80).

For example, combined genomic and epigenomic studies have revealed that certain metabolic diseases, such as Prader-Willi syndrome, are not solely caused by genetic mutations but also by epigenetic dysregulation of imprinted genes. These insights have paved the way for epigenetic therapies that target these regulatory mechanisms (81).

Metabolomics, which involves the comprehensive analysis of metabolites, complements rWGS by linking genetic variants to metabolic phenotypes. For instance, untargeted metabolomic profiling can identify biochemical signatures associated with inborn errors of metabolism and neurometabolic disorders, providing functional evidence to validate pathogenic variants identified by rWGS. Transcriptomics further enhances variant interpretation by linking genetic mutations to disrupted gene expression patterns (82, 83).

An integrated omics approach was recently demonstrated in a study on mitochondrial disorders, where rWGS identified pathogenic variants, transcriptomics confirmed aberrant gene expression, and metabolomics revealed downstream metabolic dysfunction. Such multi-omics integration has the potential to improve diagnostic accuracy and identify novel therapeutic targets for metabolic diseases (84).

### 6.2 AI and machine learning in rWGS

The vast amount of data generated by rWGS presents challenges in interpretation, particularly for rare variants and complex phenotypes. Artificial intelligence and machine learning (19) are increasingly being used to enhance variant prioritization, streamline clinical interpretation, and improve diagnostic yield.

AI-driven tools such as DeepVariant, Emedgene, and ClinPhen leverage machine learning algorithms to identify and prioritize clinically significant variants. By analyzing vast genomic datasets, these tools can detect patterns that are difficult for traditional bioinformatics pipelines to discern. For instance, ML models trained on phenotypic and genomic data can rank variants based on their likelihood of pathogenicity, dramatically reducing the time required for manual curation (85).

In metabolic diseases, AI is particularly useful for resolving VUS. For example, AI models that integrate gene expression and protein interaction networks can predict the functional impact of rare variants, helping to distinguish pathogenic mutations from benign polymorphisms. AI is also being applied to automate the interpretation of rWGS results in clinical settings. Natural language processing (NLP) tools can extract phenotypic data from electronic health records (EHRs) and map it to standardized ontologies, such as the Human Phenotype Ontology (HPO). This phenotypic information is then cross-referenced with genomic findings to generate diagnostic reports in real time (86). By automating variant annotation and prioritization, AI has the potential to reduce turnaround times, improve diagnostic consistency, and enable the broader adoption of rWGS in clinical practice. A 2023 study by Peterson et al. introduced an NLP-based pipeline that extracted phenotypic data from electronic medical records and successfully prioritized sick newborns for rWGS. Their system reduced triage time and increased diagnostic yield, showing how machine learning not only accelerates variant interpretation but also optimizes patient selection—critical for efficient resource use in clinical genomics (86).

### 6.3 Portable sequencing and decentralized labs

The development of portable sequencing technologies and decentralized laboratory models is transforming the accessibility and utility of rWGS, particularly in resource-limited settings and emergency situations. Portable sequencing devices, such as Oxford Nanopore's MinION and Flongle, allow for on-site DNA sequencing without the need for large laboratory infrastructure. These devices are lightweight, cost-effective, and capable of generating real-time sequencing data, making them ideal for use in field hospitals, rural clinics, and disaster zones (87, 88). In emergency situations, such as neonatal metabolic crises or infectious disease outbreaks, portable sequencing can provide rapid genetic insights to guide immediate interventions (89, 90, 100). A recent NICU outbreak of Serratia marcescens demonstrated that portable nanopore sequencing could quickly identify circulating clonal strains and their antimicrobial resistance genes. These insights, available within the first month of the outbreak alarm, supported timely containment and patient management efforts (89). Portable rWGS has been used to diagnose inborn errors of metabolism within hours, enabling timely treatment and improving outcomes. For instance, pulsed rapid whole-genome sequencing achieved a confirmed genetic diagnosis in two neonates in retrospective analyses within 15 and 18 h, respectively (90).

Decentralized laboratory networks, supported by cloud-based bioinformatics platforms, are further enhancing the scalability of rWGS. These models allow sequencing to be performed locally, while data analysis and interpretation are centralized through remote servers. This approach reduces the need for specialized personnel on-site and facilitates the sharing of genomic expertise across institutions (87). Efforts to establish decentralized genomic networks, such as the Global Alliance for Genomics and Health (GA4GH), are critical for democratizing access to rWGS and ensuring equitable healthcare delivery worldwide (91).

### 6.4 Expanding clinical indications

Although rWGS has been primarily applied to rare pediatric and metabolic disorders, its clinical indications are rapidly expanding to include adult-onset metabolic conditions, common diseases, and broader preventive applications. rWGS is increasingly being used to diagnose adult-onset metabolic disorders, such as mitochondrial myopathies and late-onset glycogen storage diseases, which may present with subtle or non-specific symptoms. Early identification of these conditions can prevent complications and guide targeted management strategies (6). For example, rWGS has been used to diagnose late-onset Pompe disease in adults presenting with unexplained muscle weakness, leading to the initiation of enzyme replacement therapy and improved functional outcomes (92).

Beyond rare diseases, rWGS is being explored for its potential to inform preventive care in common metabolic disorders, such as type 2 diabetes and non-alcoholic fatty liver disease (NAFLD). By identifying genetic risk factors, rWGS can enable personalized lifestyle modifications and early interventions to mitigate disease progression (93, 94).

The use of rWGS in population-level screening programs, such as newborn screening and carrier screening, is also expanding. Advances in sequencing speed and cost reduction are making it feasible to screen for a broader range of conditions, including those without established biochemical markers (9).

### 6.5 Gene therapies and CRISPR applications

The integration of rWGS with gene-editing technologies, such as CRISPR-Cas9, represents a paradigm shift in the treatment of metabolic diseases. By providing precise genetic diagnoses, rWGS enables targeted gene-editing interventions tailored to individual patients (95).

rWGS is critical for identifying the specific mutations that drive metabolic diseases, which is the first step in designing gene-editing therapies. For example, in lysosomal storage disorders such as Hurler syndrome, rWGS can pinpoint mutations in the IDUA gene, enabling the development of CRISPR-based therapies to correct the underlying defect (96).

Gene-editing technologies are also being combined with rWGS to correct pathogenic variants ex vivo, followed by the reintroduction of edited cells into the patient. This approach is being explored in clinical trials for metabolic diseases (97).

rWGS is also guiding the development of novel gene therapies that target metabolic diseases at the molecular level. For instance, AAV (adeno-associated virus)-mediated gene therapy has shown promise in conditions such as ornithine transcarbamylase (OTC) deficiency, Fabry disease, and spinal muscular atrophy (98). By identifying eligible patients and tracking treatment responses, rWGS plays a crucial role in advancing these therapies (99).

It is important to acknowledge that, in some cases, establishing a molecular diagnosis may not directly impact acute patient management. This limitation is particularly relevant for inherited metabolic disorders without targeted therapies, where clinical care remains supportive irrespective of the precise molecular etiology. Nonetheless, achieving a definitive diagnosis provides substantial benefits, including ending the diagnostic odyssey, offering prognostic clarity, enabling family planning through genetic counseling, and facilitating patient enrollment in emerging therapeutic trials. Thus, even in scenarios where immediate management is unchanged, molecular diagnosis carries enduring clinical and research value.

## 7 Conclusion

rWGS has emerged as a transformative innovation in the diagnosis and management of metabolic diseases. By enabling comprehensive genetic analysis within a compressed timeline, rWGS addresses many of the challenges associated with traditional diagnostic methods, including lengthy diagnostic time, limited scope, and inefficiency in detecting rare or complex genetic variants. Metabolic diseases often present as medical emergencies, particularly in neonates and children, where delays in diagnosis can lead to irreversible damage or death. Traditional diagnostic approaches, which rely on sequential biochemical and imaging studies, frequently fall short due to their narrow scope and prolonged time to results. rWGS has revolutionized this paradigm by offering a single, comprehensive test capable of identifying pathogenic variants across the entire genome. Governments, healthcare organizations, and researchers must work together to scale rWGS globally and integrate it into routine care. This includes investing in infrastructure, training, and policy development, as well as fostering international collaborations to share expertise and resources. Only through these concerted efforts can we ensure that the benefits of rWGS are accessible to all, transforming the diagnosis and treatment of metabolic diseases on a global scale.
